# Lectures and Examinations

**Published:** 1908-09-05

**Authors:** 


### LECTURES AND EXAMINATIONS.

A character in a work of fiction describes modern medicine as a hotch-potch of art, science, literature, and philosophy, with an admixture of arrant humbug. The influence of each of these ingredients (except, indeed, the last) can certainly be traced in every medical curriculum. Accordingly, these courses of study are far from being easy ones. Medicine, the most exacting of the professions as regards the amount of special knowledge and training needed by its followers, has its distinction continually increased by the rapid march of science. The consequent urgency and difficulty of certain present-day problems of medical education have been made evident by the time and talent devoted to recent discussions of this subject in its practical and other aspects.

Intrinsically, however, the present system of medical instruction cannot be much changed, at all events within the next decade or so, although possibly a good deal may happen to schedules and timetables. Lecturing, too, may be made less pedantic, and a nearer approach in profitableness and actuality to the demonstrations which supplement it; for, in spite of brilliant exceptions—the writer remembers a none too likely audience being stirred one cold winter's morning to unpremeditated and hearty applause by masterly treatment of a subject—these two forms of teaching have a tendency to stand to one another in the relation which Sydney Smith is said to have attributed to the duties of bishop and curate, and to have illustrated by the homely metaphor of the parlour and kitchen pokers. Again, examinational tests may be revised so as to bring out better a higher faculty than the purely literary one in its least desirable application. But such tasks are very difficult, even when the heartiest welcome is extended to reformers.

It is, therefore, of considerable importance for those who have to take things as they are—namely, the medical aspirant and especially his responsible friends, to have clearly in view from the first the exact professional position aimed at; and to judge beforehand, by the criterion of easily ascertainable results, as to the best means of gaining it. High qualifications mean stiff examinations, and high qualifications go for nearly everything in the struggle for appointments, although worldly shrewdness, amiable personal qualities, and practical ability count for much in private practice. To qualify for the more ambitious career, special tuition and preparation are advantageous. These are much more readily available at some schools than at others.

In the case of the medical profession, the antithesis hardly holds good that academic success is due to intellect, and success in after life, to character. A good deal of determination, for instance, is needed on the part of the student, generally at a pleasure-loving age and not long freed from the compulsion of school life, to render faithfully the almost daily tale of studies, and to face the prospect of repeated and severe tests of progress, which are demanded of him for some years if he aspires to write himself M.D. of London.

Moreover, for the attainment of even the highest medical degrees a combination of intellect and strength of character both a little above the average is required rather than exceptional quality of the former; if this double endowment be reinforced with social and pecuniary resources it may carry its possessor very far indeed. But for the encouragement of the modest beginner one may add that it is with medical study as with most human undertakings—the first step is by far the most difficult one. None but a born scientific worker can find some of the early non-professional subjects interesting in comparison with clinical work; and without being sure of a taste for clinical work few would take up medicine at all. Even the keenness of the individual named may be tried by the necessary but irksome effort of memorising crabbed anatomical terms. The first two years are a critical time with the student of medicine. It is a common observation that those who pass the examinations which generally conclude this period nearly always go on to qualify. Failure to reach the goal of qualification is accounted for in most cases by initial distaste or lack of perseverance. It is often at an early stage of the curriculum, too, that the relatively less successful begin to drop behind their contemporaries.

By the time, then, that the student has won the freedom of the hospital ward, he is in some measure a picked man. He is sure to have had plenty of opportunity to exercise his powers of mental assimilation, and he has shown himself capable of evincing the possession of the knowledge he has gathered. Whether an undergraduate of a resident or non-resident university, or a candidate for a medical diploma, he now enters on that part of his training which is the most essential. Oral instruction, as might be expected with a form of teaching founded on the personal relationship of master and pupil, now largely supplants text-books, at any rate at first, before the date of the final examinations has begun to loom up on the calendar. This instruction is generally excellent in quality and much appreciated, for clinical lectures and bedside demonstrations are exercises which suit the national genius very well.

